# Influence of the structural components of artificial turf systems on impact attenuation in amateur football players

**DOI:** 10.1038/s41598-019-44270-8

**Published:** 2019-05-23

**Authors:** Javier Sánchez-Sánchez, Ana M. Gallardo-Guerrero, Antonio García-Gallart, Juan Antonio Sánchez-Sáez, José L. Felipe, Alberto Encarnación-Martínez

**Affiliations:** 1 Universidad Europea de Madrid, School of Sport Sciences, Villaviciosa de Odón-Madrid, 28670 Spain; 2 Universidad Católica de Murcia, Faculty of Sports, 30107 Guadalupe-Murcia, Spain; 3 Department of Physical Education and Sports, University of Valencia, Gascó Oliag St., 3, Valencia, 46010 Spain

**Keywords:** Bone quality and biomechanics, Risk factors

## Abstract

The purpose of this research was to evaluate the influence of the structural components of different 3rd generation artificial turf football field systems on the biomechanical response of impact attenuation in amateur football players. A total of 12 amateur football players (24.3 ± 3.7 years, 73.5 ± 5.5 kg, 178.3 ± 4.1 cm and 13.7 ± 4.3 years of sport experience) were evaluated on three third generation artificial turf systems (ATS) with different structural components. ATS were composed of asphalt sub-base and 45 mm of fibre height with (ATS1) and without (ATS2) elastic layer or compacted granular sub-base, 60 mm of fibre height without elastic layer (ATS3). Two triaxial accelerometers were firmly taped to the forehead and the distal end of the right tibia of each individual. The results reveal a higher force reduction on ATS3 in comparison to ATS1 (+6.24%, CI95%: 1.67 to 10.92, ES: 1.07; *p* < 0.05) and ATS2 (+21.08%, CI95%: 16.51 to 25.66, ES: 2.98; *p* < 0.05) elastic layer. Tibia acceleration rate was lower on ATS3 than ATS1 (−0.32, CI95%: −0.60 to −0.03, ES: 4.23; *p* < 0.05) and ATS2 (−0.35, CI95%: −0.64 to −0.06; ES: 4.69; *p* < 0.05) at 3.3 m/s. A very large correlation (*r* = 0.7 to 0.9; *p* < 0.05) was found between energy restitution and fibre height in both head and tibial peak acceleration and stride time. In conclusion, structural components (fibre height, infill, sub-base and elastic layer) determine the mechanical properties of artificial turf fields. A higher force reduction and lower energy restitution diminished the impact received by the player which could protect against injuries associated with impacts compared to harder artificial turf surfaces.

## Introduction

Football is a sport characterized by high-intensity periods and recovery phases alternating with frequent accelerations and sudden changes of directions^1^. The development of new technologies in this sport has allowed the detection of the influence of external variables on the performance in these actions and the safety of the players^2^. In this way, the sport surface has been identified as an external factor that affects football players’ performance and risk of injury^3,4^. Several studies have analysed the differences between natural and artificial turf in football^5,6^. However, Sánchez-Sánchez *et al*.^7^, revealed a mechanical heterogeneity between the different 3^rd^ generation systems of artificial turf. In fact, previous studies showed that the differences between various systems of artificial turf can be even higher than those between natural grass and artificial turf ^8^.

The evolution of the structural components of 3rd generation football fields has resulted in a great diversity of structural combinations between the type of fibre, the filling material, the elastic layer installed, or the selected sub-base^9^. In this sense, previous studies have shown that these structural combinations influence on the mechanical behavior, the response to the intensity of use and the level of maintenance required^10,11^. Within the support structure, the type of sub-base selected significantly determines the vertical loads and the properties of impact absorption, energy of restitution and standard vertical deformation of the playing surface^11–15^.

The systems of artificial turf composed by a sub-base of compacted gravel without a shockpad have proven a force reduction and vertical deformation below the limits specified in the normative^9,12,16^. On the other hand, the elastic layer has demonstrated a great ability to maintain the mechanical properties of the playing surface^9^ ensuring optimal safety and functionality of the surface. In relation to the surface components, sand and rubber are direct determinants of the mechanical performance of the surface^14,17–19^. A change in the type or morphology of rubber generates differences in the mechanical behaviour of football fields of artificial turf^10,14^. In this sense, Fleming, Forrester, and McLaren^17^, revealed that the density of the rubber and its level of compaction cause changes in the absorption of impacts. These authors demonstrated that the influence of the carpet fibres in resistance to vertical load is minimal. It is thought that the fibres will act to resist horizontal strain in the infill layer and thus vertical deformation is reduced.

Therefore, a good combination of structural components is decisive for the shock absorption of the impacts exerted by football players. Each strike of the foot with the ground produces an impact or shock, which is absorbed throughout the body from the foot to the head^20–22^. A limited dissipation of these impacts can produce suboptimal performances, overloads and injuries^23^. Due to this, the analysis of the magnitude and attenuation of the shock wave have acquired a very important role in the scientific community^24^. This analysis using accelerometry can determine the evolution of the signal in time, or analyze the frequency components, allowing to distinguish between movements of low frequencies due to own human movement and resonance of high frequencies related to the severity of impacts^25^.

Several researches have analysed the effect of artificial turf system on the mechanical properties of the fields^8^, the performance of players^7^ or their perceptions^26^. According to the risk of injury of the players, Zanetti, Bignardi, Franceschini and Audenino^19^ evidenced different peak vertical accelerations in two artificial turf system with different infill material. However, there are no studies that relate the pile height and the effect of the shockpad on the mechanical parameters and the impacts received by players. Therefore, the purpose of this research has been to evaluate the influence of the structural components of different 3rd generation artificial turf football field systems on the biomechanical response of impact attenuation on amateur football players.

## Results

The results show significant differences in the mechanical behavior of the artificial turf systems analysed (p < 0.05, Table 1). ATS3 shows higher RF than ATS1 (+6.24%, CI95%: 1.67 to 10.92, ES: 1.07) and ATS2 (+21.08%, CI95%: 16.51 to 25.66, ES: 2.98). Similarly, ATS3 revealed a higher StV of the fibre than ATS1 (+2.74 mm, CI95%: 1.97 to 3.51, ES: 2.17) and ATS2 (+5.91 mm, CI95%: 5.14 to 6.68, ES: 5.58). In relation to ER, ATS3 showed significantly lower values than ATS1 (−5.66%, CI95%: −9.50 to −1.82, ES: 1.37) and ATS2 (−3.88%, CI95%: −7.62 to −0.04, ES: 0.60).

**Table 1:** Spatio-temporal variables, impacts in time domain and frequency on three surfaces of artificial turf at different speeds in football players.

	ATS 1 (1)			ATS 2 (2)			ATS 3 (3)		
	3.3 m/s (12 km/h)	4 m/s (14.4 km/h)	Vmax	3.3 m/s (12 km/h)	4 m/s (14.4 km/h)	Vmax	3.3 m/s (12 km/h)	4 m/s (14.4 km/h)	Vmax
Spatio-temporal									
TPTA (ms)	21.06 ± 1.96^c^	20.98 ± 2.00^c^	24.00 ± 3.07^c^	21.15 ± 1.53^c^	21.66 ± 1.31^c^	22.97 ± 1.61^c^	28.73 ± 2.05^a,b^	29.71 ± 1.78^a,b^	31.43 ± 4.56^a,b^
TPHA (ms)	21.17 ± 1.95^c^	21.10 ± 2.00^c^	24.06 ± 3.07^c^	21.29 ± 1.50^c^	21.77 ± 1.32^c^	23.03 ± 1.60^c^	28.88 ± 2.06^a,b^	29.85 ± 1.78^a,b^	31.53 ± 4.59^a,b^
Stride Time (ms)	705.35 ± 40.42^c^	684.68 ± 36.58^c^	552.35 ± 57.68^c^	694.63 ± 33.15^c^	679.32 ± 45.41^c^	543.56 ± 43.22^c^	983.53 ± 68.32^a,b^	947.50 ± 34.91^a,b^	735.00 ± 11.94^a,b^
Stride length (m)	2.33 ± 0.15	2.74 ± 0.16	3.06 ± 0.64	2.32 ± 0.14	2.84 ± 0.74	3.16 ± 0.59	2.36 ± 0.14	2.68 ± 0.11	3.31 ± 0.07
Stride frequency (Hz)	1.80 ± 0.11	1.86 ± 0.18	2.29 ± 0.18	1.81 ± 0.13	1.84 ± 0.11	2.39 ± 0.19	1.77 ± 0.11	1.82 ± 0.07	2.47 ± 0.22
Time impacts									
Tibia Acc Rate (g/ms)	0.56 ± 0.26^c^	0.76 ± 0.30	1.26 ± 0.20	0.60 ± 0.39^c^	0.75 ± 0.46	1.54 ± 0.46	0.25 ± 0.07^a,b^	0.57 ± 0.41	1.39 ± 0.09
Head Acc Rate (g/ms)	0.09 ± 0.03	0.12 ± 0.06^c^	0.32 ± 0.16^c^	0.09 ± 0.04	0.09 ± 0.04	0.26 ± 0.09	0.04 ± 0.01	0.04 ± 0.01^a^	0.21 ± 0.07^a^
Satt (%)	63.00 ± 4.97	64.25 ± 4.40	62.13 ± 13.35	63.22 ± 6.68	63.69 ± 3.79	66.19 ± 5.02	67.36 ± 7.17	67.19 ± 4.10	63.86 ± 6.87
TPM (g)	9.68 ± 1.79	10.79 ± 2.10	16.01 ± 1.73	9.10 ± 2.10	10.15 ± 2.23	14.34 ± 1.89	8.63 ± 1.87	10.42 ± 2.33	14.16 ± 1.90
HPM (g)	2.74 ± 0.49^c^	2.95 ± 0.59	4.06 ± 0.65	2.77 ± 0.49^c^	2.95 ± 0.52	3.88 ± 0.75	2.16 ± 0.46^a,b^	2.51 ± 0.54	3.78 ± 0.38
Frequency impacts									
TSMlow (g^2^/Hz)	0.34 ± 0.08	0.50 ± 0.15	4.16 ± 1.34	0.31 ± 0.08	0.48 ± 0.14	4.56 ± 1.35	0.37 ± 0.10	0.55 ± 0.06	4.00 ± 1.04
TSMhigh (g^2^/Hz)	0.55 ± 0.22	0.75 ± 0.27	2.57 ± 0.90	0.44 ± 0.15	0.63 ± 0.17	2.41 ± 1.10	0.41 ± 0.13	0.59 ± 0.22	2.17 ± 0.17
HSMlow (g^2^/Hz)	0.44 ± 0.08	0.47 ± 0.10	0.16 ± 0.14	0.42 ± 0.08	0.46 ± 0.10	0.15 ± 0.08	0.39 ± 0.06	0.44 ± 0.08	0.12 ± 0.05
HSMhigh (g^2^/Hz)	0.06 ± 0.03	0.06 ± 0.03	0.31 ± 0.17	0.05 ± 0.03	0.06 ± 0.02	0.25 ± 0.13	0.05 ± 0.02	0.06 ± 0.03	0.22 ± 0.11
ATTlow (dB)	−17.20 ± 18.41	−21.70 ± 16.18	−84.21 ± 28.33	−18.11 ± 18.41	−20.95 ± 14.85	−89.93 ± 18.47	−16.02 ± 7.94	−22.61 ± 11.96	−90.82 ± 13.45
ATThigh (dB)	−125.02 ± 23.07	−148.75 ± 36.24	−118.79 ± 26.59	−122.07 ± 26.66	−137.91 ± 22.61	−115.96 ± 28.21	−134.35 ± 29.28	−139.86 ± 19.21	−109.87 ± 15.43

*Positive values for ATTlow, high indicate a gain in signal power whereas negative values indicate attenuation of signal power. ^a,b,c^Significant differences (*p* < 0.05) with the Artificial Turf System indicated (ATS). TPTA: Time to Tibial Peak Acceleration; TPHA: Time to Head Peak Acceleration; Satt: Shock Attenuation; TPM: Tibial Peak Acceleration Magnitude; HPM: Head Peak Acceleration Magnitude; TSM: Tibial Signal Magnitude; HSM: Head Signal Magnitude; ATT: Shock Attenuation.

In relation to the spatio-temporal variables, the players showed higher TPTA, TPHA and Stride Time in ATS3 compared to the rest of surfaces (*p* < 0.05, Table 1). On the other hand, ATS3 revealed a lower Tibia Acc Rate than ATS1 (−0.32, CI95%: −0.60 to −0.03, ES: 4.23) and ATS2 (−0.35, CI95%: −0.64 to −0.06; ES: 4.69) at 3.3 m/s (12 km/h). Head Acc Rate results showed higher values in ATS1 compared to ATS3 at 4 m/s (14.4 km/h) (+0.08, CI95%: 0.01 to 0.11, ES: 4.38) and Vmax (+0.11, CI95%: 0.01 to 0.21 ES: 4.75). Finally, a higher HPM was obtained in players over ATS1 (+0.58, CI95%: 0.15 to 1.02, ES: 5.11) and ATS2 (+0.61, CI95%: 0.18 to 1.05, ES: 5.36) compared to ATS3.

Both large and very large significant correlations were found between ER and fibre height with TPTA (*r* = −0.73; *r* = 0.79), TPHA (*r* = −0.74; *r* = 0.80, respectively), Stride Time (*r* = −0.73; *r* = 0.82) and Stride Frequency (*r* = 0.60; *r* = −0.69), respectively. On the other hand, StV revealed a positive large correlation with TPTA (*r* = 0.60), TPHA (*r* = 0.60) and Stride Time (*r* = 0.65). Also, this variable showed a negative large correlation with Stride Frequency (*r* = −0.58; Table 2).

**Table 2:** Correlation between mechanical properties of artificial turf and spatial-temporal variables and impact of soccer players.

	TPTA	TPHA	Stride Time	Stride length	Stride Frequency	Tibia AccRate	Head AccRate	SAtt
FR (%)	0.47**	0.47**	0.54**	0.20**	−0.49**	−0.24**	−0.12	0.05
StV (mm)	0.60**	0.60**	0.65**	0.25**	−0.58**	−0.26**	−0.17*	0.08
ER (%)	−0.73**	−0.74**	−0.73**	−0.36**	0.60**	0.16*	0.29**	−0.18**
Rubber infill (mm)	0.33**	0.33**	0.40**	0.13	−0.37**	−0.22**	−0.06	0.01
Fibre Height (mm)	0.79**	0.80**	0.82**	0.37**	−0.69**	−0.24**	−0.28**	0.17*
	**TPM**	**HPM**	**TSMlow**	**TSMhigh**	**HSMlow**	**HSMhigh**	**ATTlow**	**ATThigh**
FR (%)	−0.08	−0.25**	−0.20**	−0.15*	0.12	−0.11	0.18**	−0.14*
StV (mm)	−0.10	−0.28**	−0.19**	−0.16*	0.10	−0.12	0.17*	−0.13
ER (%)	0.13	0.23**	0.03	0.10	0.06	0.07	−0.03	−0.003
Rubber infill (mm)	−0.05	−0.21**	−0.19**	−0.13	0.14*	−0.10	0.18**	−0.14*
Fibre Height (mm)	−0.14*	−0.30**	−0.12	−0.15*	0.02	−0.11	0.11	−0.07

*p < 0.05; **p < 0.01; FR: Force Reduction; StV: Vertical Deformation; ER: Energy Restitution. TPTA: Time to Tibial Peak Acceleration; TPHA: Time to Head Peak Acceleration; Satt: Shock Attenuation; TPM: Tibial Peak Acceleration Magnitude; HPM: Head Peak Acceleration Magnitude; TSM: Tibial Signal Magnitude; HSM: Head Signal Magnitude; ATT: Shock Attenuation.

## Discussion

This is the first study that analyzes the biomechanical response of impact attenuation taking into account the mechanical parameters of artificial turf. The main finding of this research was the significant association between the mechanical properties of artificial turf fields (shock absorption, standard vertical deformation and energy of restitution), the structural parameters of the field and the impact attenuation in amateur football players.

The analysis of the mechanical parameters of the selected fields evidenced the effect of the support structure on the mechanical behavior of artificial turf football fields. Previous studies revealed the importance of an elastic layer in the conservation of mechanical properties over time^27^, as well as in the attenuation of the intensity of the impacts exerted by players^9^. The present study revealed a difference of 30.32% in the force reduction of the surface between two equal artificial grass systems (ATS1 and ATS2) after the incorporation of an elastic layer on an asphalt sub-base. However, the field with greater force reduction and vertical deformation of the fibre did not incorporate this component (ATS3). A sub-base of compacted gravel, a type of SBR rubber and a higher height and quality of the fibre were the causes of this result^7,10,28^. For this reason, the correlation analysis revealed a very large positive influence of the pile length on TPTA, TPHA and stride time, although an artificial turf system with lower fibre height can obtain similar values of damping and attenuation of impacts through an adequate combination of the structural components^11^.

A higher fibre height requires a greater amount of infill that compensates the elasticity of the elastic layer incorporated in ATS1^29^. On the other hand, Burillo, Gallardo, Felipe and Gallardo^28^ evidenced a greater force reduction in artificial turf fields that incorporated a SBR infill instead of thermoplastic, confirming the differences found in the present study. Finally, a well-compacted sub-base of compacted granular has a greater capacity of attenuation of the impacts received on the surface structure compared to a sub-base of asphalt, increasing the force reduction of the system, independent of the elastic layer. The comparison of the mechanical properties of the three artificial turf systems also showed a higher energy restitution in ATS1. This variable measures the energy returned to the player from the surface after an impact^16^. This property has been shown to be decisive in the physical performance of the players, improving time and sprint speed^30^, as well as running economy and fatigue^31,32^ on those fields with the highest restitution energy. However, the results of this study have shown a very large negative correlation (*r* > 0.7) of this parameter in the time between contact time and peak acceleration of the tibia and head and time of stride, being the mechanical property more related to the attenuation of impacts on artificial turf systems.

During running, the impacts are attenuated internally by passive structures such as bones, cartilages and ligaments, by active movements and by external components such as footwear and surface^33,34^.

This study reveals that ATS3 (compacted granular sub-base, 60 mm of fibre length and higher quantity of infill) is the softest surface with fewer impacts when football players run. These results coincide with previous researches^35^ where the type of sub-base determined the hardness of the surface, being the sand a soft surface with the lowest number of acceleration registered.

The high distances reached during football matches generate a lot of impacts and the sprint performance of the football players should be evaluated in the light of previous studies^36–38^. It has been demonstrated that the severity of impact can be higher as fatigue increases^39,40^ and it could also be related with stress injuries^34,39–41^. Moreover, the incidence of football match injuries increases over time towards the end of both the first and second halves where it has been speculated that fatigue might be an explanation for it^42^. With this in mind, artificial turf surfaces with more soft and flexible structural characteristics could help to diminish the severity of impacts, being more relevant when the body’s ability to receive the impacts is diminished by fatigue towards the end of each half, where there is a greater risk of injury.

However, spite these benefits in the impact attenuation, football fields that are too soft increase the injury risk derived from an early muscle fatigue^9,43^. Related to this, Williams, Trewartha, Kemp, Michell and Stokes^44^ showed how muscle soreness on artificial turf was higher over the 4 days following a match in elite English Premiership Rugby Union players compared to natural grass. This coincides with Poulos *et al*.^45^. These authors after evaluating 99 professional football players of the main football league of the United States and Canada (Major League Soccer) indicated that 97% of them perceived greater muscle and joint soreness playing on artificial turf.

Recently, it has been suggested that artificial turf causes higher rate of foot injuries, especially midfoot and toe fractures including dislocations^46^. These type of injuries are considered overuse injuries, which were higher on World Rugby certified third generation artificial turf compared to natural grass in elite rugby players^47^. Thus, overuse injuries and playing surface could be linked due to different traction and cushioning characteristics between surfaces^47^. Ranson, George, Rafferty, Miles and Moore^46^ suggested that the highest foot injuries could be related to the greater traction, stiffness and rotational torque shown on artificial turf in comparison with natural grass. But these greater demands maybe also could occur between different artificial turf with different hardness, thickness or amount of infill, similar to this research. In this study, ATS1 and ATS2 shown a lower force reduction and vertical deformation in comparison to ATS3. According to the results, the stiffness of the surface influence on the impact attenuation and could determine the kinetic and kinematic movement of the football players, causing different physiological and metabolic responses^44,48^ where each playing surface could have its own characteristics of injury patterns depending on its structural characteristics^47,49^.

Because of that, the results of the present study must be taken with caution. Softer artificial turf surfaces could diminish the impact received and protect against injuries associated with the impacts compared to harder artificial turf surfaces but could also lead to greater physiological demand and earlier fatigue that would increase the risk of overuse injuries associated with fatigue. Thus, softer or harder artificial turf surfaces will have their own advantages and disadvantages that will be opposite to each other. Therefore, future lines of research are necessary to achieve artificial turf surfaces by combining or creating structural components with an optimum level of firmness that protect against impacts as do softer surfaces and generate physiological requirements similar to those provided by harder surfaces, avoiding early fatigue and protecting against overuse injuries.

## Methods

### Participants

A total of 12 healthy men amateur football players (24.3 ± 3.7 years, 73.5 ± 5.5 kg, 178.3 ± 4.1 cm and 13.7 ± 4.3 years of sport experience) agreed to participate and gave informed written consent. Inclusion criteria included no history of lower extremity injuries within the last year. The study protocol was approved by the Local Ethics Committee (Toledo Hospital). All research was performed in accordance with the Code of Ethics of the World Medical Association (Declaration of Helsinki) and all of the participants signed an informed consent form in which the test procedures and possible risks were explained.

### Study design

Two triaxial accelerometers (AcelSystem, Spain) (sampling frequency: 300 Hz, measuring range: up to 16 g, and mass; 2.5 grams) were firmly taped to: a) the forehead; and b) the distal end of the right tibia (Distal)^24^. Three third generation artificial turf systems (ATS) were chosen according to the structural components. The manufacturer of the ATS selected was the same (Mondo Ibérica S.A., Zaragoza, Spain). All the pitches were similar in state; the age (<5 years old), wear (<35 h/week) and maintenance was similar in the ATS of the study. The length of the fibre, the type of sub-base, the quantity of the infill and the elastic layer were different in the artificial turf fields (Table 3).

**Table 3:** Characteristics of the artificial turf systems (ATS) selected.

Characteristics	ATS 1	ATS 2	ATS 3
Fibre			
Model	3NX	3NX	4NX
Fibre material	Polyethylene	Polyethylene	Polyethylene
Fibre type	Monofilament	Monofilament	Monofilament
Pile height	45 mm	45 mm	60 mm
Yarn Thickness	250 µ	250 µ	400 µ
Tufting	In line	In line	In line
Gauge	5/8	5/8	5/8
Pile weight	1119 g/m²	1119 g/m²	1427 g/m²
Stitches/m²	8750	8750	8750
Dtex	12000	12000	12000
Infill			
Sand material	Quartz	Quartz	Quartz
Granulometry	0.3–0.8 mm	0.3–0.8 mm	0.3–0.8 mm
Quantity	20 Kg/m²	20 Kg/m²	16 Kg/m²
Rubber material	Thermoplastic vulcanised	Thermoplastic vulcanised	SBR
Granulometry	1–3 mm	1–3 mm	0.5–2.5 mm
Quantity	9 Kg/m²	9 Kg/m²	17 Kg/m²
Support structure			
Sub-base material	Asphalt	Asphalt	Compacted granular
Elastic layer	Yes	No	No
Elastic layer thickness	17 mm	—	

ATS: Artificial Turf System.

A linear running test on each of the pitches, with at least of a 48 hours separation between, was measured in three running speeds: 3.33 m/s (12 km/h)^22^ (slow), 4 m/s (14.4 km/h) (medium), and at maximum speed in 20 m straight (fast). A separation of at least of 48 h between pitches was left in order to avoid the fatigue effects.

A 12 minutes standard warm up was done before the running rhythms familiarization phase. Then, the participants tested the running rhythms established except the maximum speed run, until they felt comfortable with the conditions of the study^20^. Three repetitions for each speed in a randomized order was done within 20 m of the middle of the pitch. Photoelectric cells (Microgate, Bolzano, Italy), positioned along 5 m in the middle of the 20 m pitch, was employed to control the speed. The accelerometer signal was registered during 5 s making this register coincide with running past the photocells to guarantee speed stability and avoid moments of initial acceleration and final deceleration of the test^20,50^. A ± 5% speed deviation was allowed during each speed condition. Otherwise, the measurement had to be carried out again. The participants returned walking slowly to the initial starting position after each repetition, and a 2 min rest time was established between repetitions in order to avoid fatigue^51^. Accelerations were collected at 300 Hz for 15 seconds at the end of each run.

It should be noted that a familiarization session was carried out. Moreover, all the tests performed out in the morning, at a similar time, in three different systems of artificial turf and with a minimum of 72 hours between them.

Data were analysed using Matlab (MathWorks, MA, USA). For the time-domain analysis, the vertical acceleration signal was filtered (Butterworth, second-order, low-pass, cut-off frequency = 50 Hz) and the tibial positive peak acceleration and the magnitude shock attenuation were calculated as reported^52^. Time to Head and tibial peak acceleration (TPHA and TPTA respectively) (time between contact time and peak acceleration), Head and tibial peak acceleration magnitude (HPM and TPM respectively) (difference between the minimum and maximum acceleration), Head and tibia acceleration rate (AccRate) (acceleration gradient between contact time and peak acceleration), and Shock attenuation (SAtt) (reduction in peak impact acceleration from the tibia to the head) were calculated for each step of the right foot^22,39,52^. Stride length (distance between successive points of heel contact of the same foot), Stride time (time of stride) and Stride frequency (number of heel contacts of the same foot per second) were also calculated by the signal of the accelerometers.

For the frequency-domain analysis, the non-filtered stance phases extracted from the time-based signal were analysed. After removing the mean and linear trends, the signals were padded with zeroes to equal 2048 data points and power spectrums were calculated for the head and the tibia^25^. Analyses of the low (3–8.5 Hz) and high (8.5–20 Hz) frequency ranges were performed to investigate the behaviour of the two local acceleration peaks occurring during running^22,25^. In order to measure the impact attenuation, a transfer function was calculated from the power spectrum of the head and tibia^25^. In summary, the following frequency-domain variables were calculated in both the low and high frequency ranges: Tibial Signal Magnitude (TSM_low_ and TSM_high_), Head Signal Magnitude (HSM_low_ and HSM_high_), and Shock attenuation (ATT_low_ and ATT_high_).

### Mechanical properties of the playing surface

The quality standards of the FIFA Quality Programme for Football Turf-Handbook of Test Methods^53^ were taken into account for the initial assessment of the mechanical properties of the chosen artificial turf systems. The variables used were the following: Energy of Restitution (ER), Force Reduction (FR) and Standard Vertical Deformation (StV). The aforementioned variables were tested in several positions as stated by FIFA Regulations (Fig. 2).

**Figure 1:**
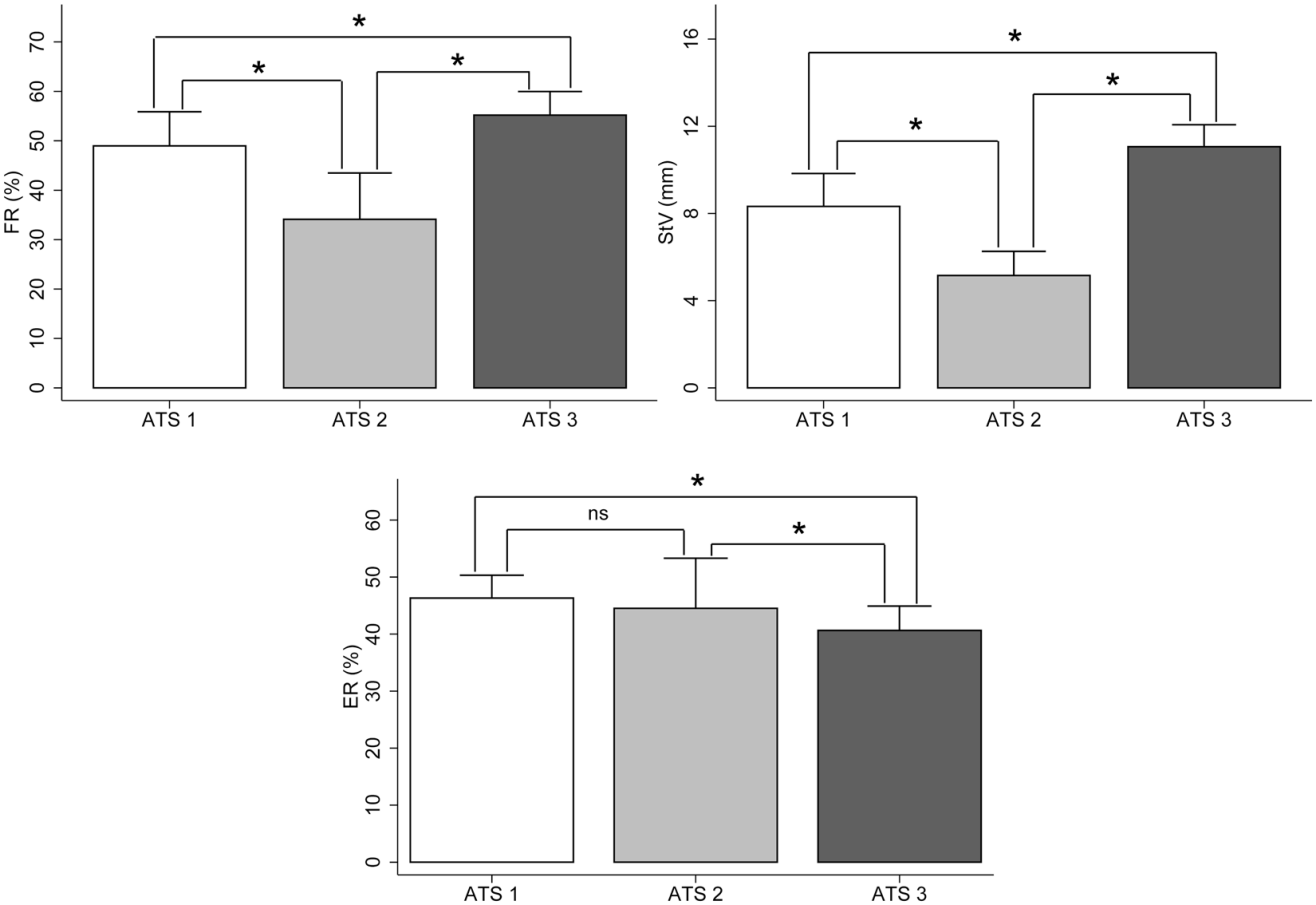
Mechanical properties of artificial turf systems analysed. *Significant differences between artificial turf systems (*p* < 0.05). ^ns^Not significant; ATS: Artificial Turf System; FR: Force reduction; StV: Vertical deformation; ER: Energy of restitution.

**Figure 2:**
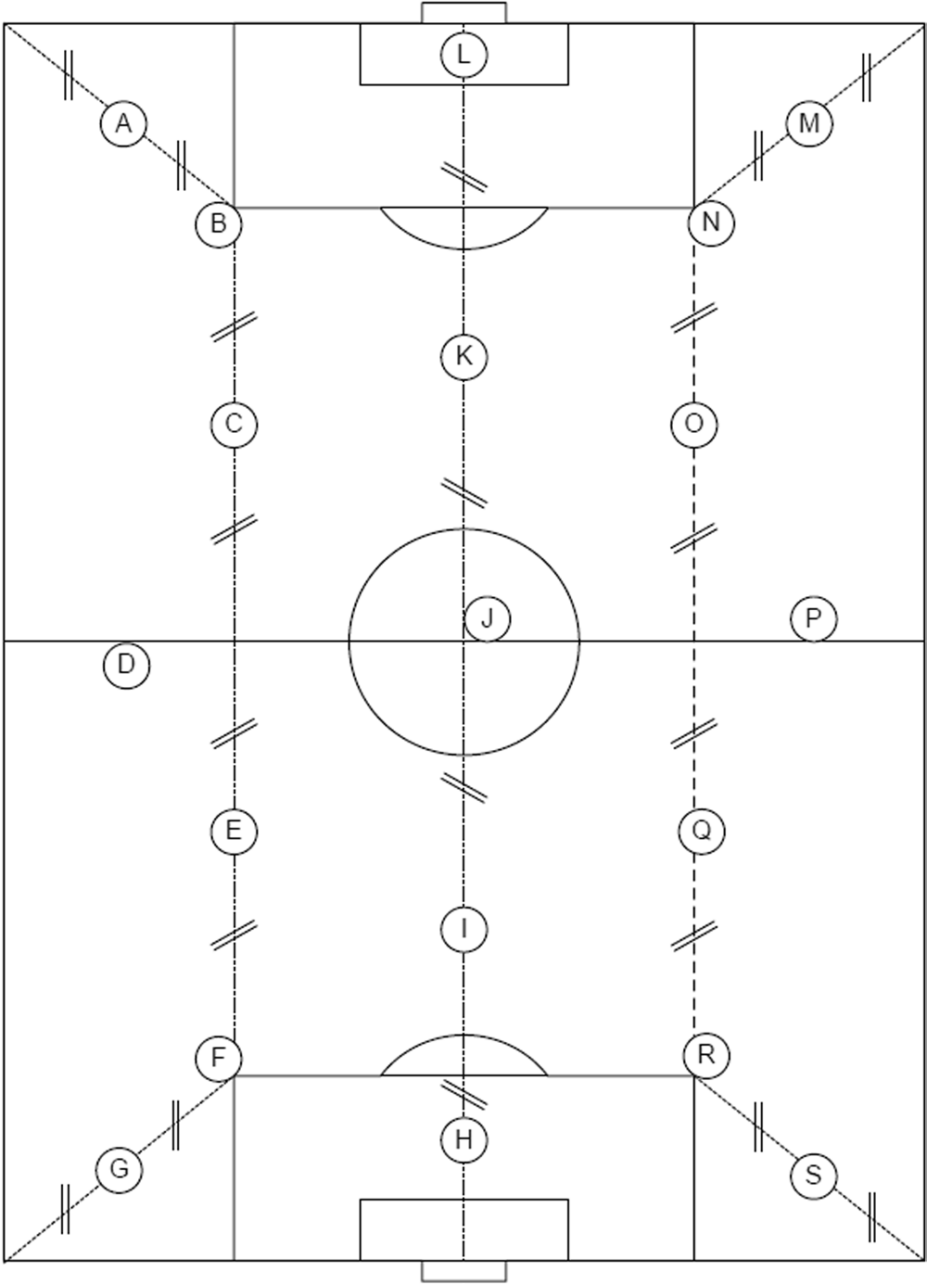
19 Field Test Positions according to FIFA requirements^53^. 15 test positions are fixed and shall be in the general positions shown. Positions – F, R, N and B may be in the positions shown or other locations selected at the discretion of the test researcher.

The conditions in which the tests were completed were dry, as well as the ranges used that were within the FIFA Quality Programme for Football Turf protocol^53^. In the following descriptions, we can observe how the mechanical property tests were taken place.

### Energy of restitution, force reduction and standard vertical deformation

Energy of Restitution (ER), Force Reduction (FR) or shock absorption and the Standard Vertical Deformation (StV) were all tested under the FIFA Test Method 04a, 05a and 13 protocols. In addition, an Advanced Artificial Athlete devise was also used. A spring with controlled stiffness was used, a mass of 20 kg (±100) was dropped from a height of 55 mm (±0.25 mm).

The Force Reduction was determined taking into account the force of impact on the concrete (reference force) in comparison with the maximum force applied on an artificial turf field. The maximum applied force was recorded using a load cell when its spring hits the artificial turf surface expressing the results as a percentage. The following equation was used to estimate the FR to a resolution of 0.01%:

$$ \% {\rm{FR}}=(1-\frac{Fmax\,}{Fref\,})\cdot 100 \% $$

In this equation the %FR is the Force Reduction which is expressed in %, followed by *Fmax* being the maximum force which is measured on the artificial turf and is expressed in N and finally the *Fref* is 6760N which is a fixed reference force using an absolute resolution of the apparatus of 0.02 kN that corresponds with the theoretical value that has been determined for concrete surfaces.

The impact produced by a falling mass onto the surface and the displacement produced after impact determined the StV. The double integration of the acceleration produced, a(t), over the interval [T1, T2] determines the displacement produced by the falling mass *Dmass* (t). Integration starts at the interval (T1) which is the moment when the highest velocity has been reached by the mass (Vmax) and then finishes at (T2) being recorded during the rebounds after the impact on the test specimen registering the maximum absolute velocity time of the mass (Vmin). Depending on the way the accelerometer has been set up, Vmax and Vmin may produce positive or negative values.

Below the StV of the test specimen determined over the time interval is as following:

$$StV={\rm{D}}mass-Dspring$$

where:

$$Dmass={\iint }_{T1}^{T2}g\,dt,\,{\rm{with}}\,Dmass=0\,{\rm{mm}}\,{\rm{at}}\,T1$$

$$Dspring=(m\cdot g\cdot {G}_{max})/{C}_{spring}$$

Above, the peak acceleration registered during impact (*Gmax*) is expressed in g. Furthermore, m is the falling mass which is expressed in kg and g is the acceleration caused by gravity (9.81 m/s²). *Cspring* is expressed in N/mm and is the spring constant. Both FR and StV were recorded in 19 different test positions (Fig. 1). A total of three impacts were produced in each position. For analysis purposes the average of the second and third impact were used.

Finally, ER was determined with the following variables:

$$ER\,=(\frac{{\rm{E}}2}{{\rm{E}}1})\cdot 100$$

where: E1 is the energy before impact [E1 = 0.5 × mV^2^ max], E2 is the energy after impact [E2 = 0.5 × mV^2^ min], Vmax is the velocity before impact at T1 in m/s, Vmin is the velocity after impact at T2 in m/s, *m* is the falling mass including spring, base plate and accelerometer, expressed in kg.

### Data analysis

Data are presented as mean ± SD. Acceleration signal was filtered with a 60 Hz low pass Butterworth filter^22^. Then, a MATLAB R2013b routine (Mathworks Inc, Natick, MA) expressly made for the study was used to calculate the variables. Two-way ANOVA and Bonferroni post-hoc test was used to analyse the differences between different surface and speed. Confidence interval of the differences (CI of 95%) was included and effect size was calculated to identify the magnitude of changes (ES; Cohen’s d). The ES was evaluated following the next criteria: ES was assessed using the following criteria: < 0.2 = trivial, 0.2–0.6 = small, 0.6–1.2 = moderate, 1.2–2.0 = large, and > 2.0 = very large^53,54^. Correlations were evaluated with the following criteria: 0 to 0.1 = trivial, 0.1 to 0.3 = small, 0.3 to 0.5 = medium, 0.5 to 0.7 = large, 0.7 to 0.9 = very large and 0.9 to 1.0 = nearly perfect^55^. SPSS 21.0 was used for the data analysis and the level of significance was established at *p* < 0.05.
